# A randomized, double-blind, placebo-controlled trial to assess safety and tolerability during treatment of type 2 diabetes with usual diabetes therapy and either Cycloset™ or placebo

**DOI:** 10.1186/1472-6823-7-3

**Published:** 2007-06-25

**Authors:** Richard E Scranton, J Michael Gaziano, Dean Rutty, Michael Ezrokhi, Anthony Cincotta

**Affiliations:** 1 Veroscience LLC, Tiverton RI, Harvard Medical School, Boston, USA; 2 Division of Aging, Brigham & Women's Hospital, Harvard Medical School, Boston, USA; 3 Everest Clinical Research Services Inc., Toronto, Canada

## Abstract

**Background:**

Cycloset™ is a quick-release formulation of bromocriptine mesylate, a dopamine agonist, which in animal models of insulin resistance and type 2 diabetes acts centrally to reduce resistance to insulin- mediated suppression of hepatic glucose output and tissue glucose disposal. In such animals, bromocriptine also reduces hepatic triglyceride synthesis and free fatty acid mobilization, manifesting decreases in both plasma triglycerides and free fatty acids. In clinical trials, morning administration of Cycloset™ either as monotherapy or adjunctive therapy to sulfonylurea or insulin reduces HbA1c levels relative to placebo by 0.55–1.2. Cycloset™ therapy also reduces plasma triglycerides and free fatty acid by approximately 25% and 20%, respectively, among those also receiving sulfonylurea therapies. The effects of once-daily morning Cycloset™ therapy on glycemic control and plasma lipids are demonstrable throughout the diurnal portion of the day (7 a.m. to 7 p.m.) across postprandial time points.

**Methods/Design:**

3,095 individuals were randomized in a 2:1 ratio into a one year trial aimed to assess the safety and efficacy of Cycloset™ compared to placebo among individuals receiving a variety of treatments for type 2 diabetes. Eligibility criteria for this randomized placebo controlled trial included: age 30–80, HbA1c ≤ 10%, diabetes therapeutic regimen consisting of diet or no more than two hypoglycemic agents or insulin with or without one additional oral agent (usual diabetes therapy; UDT). The primary safety endpoint will test the hypothesis that the rate of all-cause serious adverse events after one year of usual diabetes therapy (UDT) plus Cycloset™ is not greater than that for UDT plus placebo by more than an acceptable margin defined as a hazard ratio of 1.5 with a secondary endpoint analysis of the difference in the rate of serious cardiovascular events, (myocardial infarction, stroke, coronary revascularization or hospitalization for or angina or congestive heart failure). Efficacy analyses will evaluate effects of Cycloset™ versus placebo on change from baseline in HbA1c, fasting glucose, body weight, waist circumference, blood pressure and plasma lipids.

**Discussion:**

This study will extend the current data on Cycloset™ safety, tolerability and efficacy in individuals with type 2 diabetes to include its effects in combination with thiazolodinediones, insulin secretagogues, metformin, alpha-glucosidase inhibitors and exogenous insulin regimens.

**Trial registration:**

*clinical trials.gov *NCT00377676

## Background

Reduced insulin-mediated glucose disposal and impaired suppression of endogenous (hepatic) glucose production are dominant metabolic features of the obese state. [1] It has been estimated that 60–80 percent of type 2 diabetic patients are obese. [2] Among representative species of all the major vertebrate classes of animals, the development of obesity and its accompanying insulin resistance has certain adaptive advantages. This adaptation improves survival in times of seasonal famine.[3] Many vertebrate species develop obesity and insulin resistance in preparation for hibernation, migration or during over-wintering periods when food availability is extremely low.[4] Extensive experimental evidence indicates that circadian neuroendocrine rhythms play a pivotal role in the development of seasonal changes in body fat stores and insulin sensitivity. Specifically, temporal changes in the interaction of two distinct circadian neural circadian oscillations, mediated in part by dopaminergic and serotonergic neurotransmitter activity, have been shown to regulate the dramatic seasonal alterations in body weight and body composition that are characteristic of all vertebrate classes from teleosts to mammals.[3] Additional data obtained in pigs and rats suggest that similar mechanisms may play a role in the development of nonseasonal (e.g., aging-related) obesity and insulin resistance.[5,6]

Bromocriptine mesylate, an ergot derivative, is a sympatholytic dopamine D2 receptor agonist that can exert inhibitory effects on serotonin turnover in the central nervous system.[7] Available evidence suggests that bromocriptine can reverse many of the metabolic alterations associated with insulin resistance and obesity by resetting central (hypothalamic) circadian organization of monoamine neuronal activities.[8] Additionally, dopamine agonist treatment reduces ventromedial, arcuate and paraventricular hypothalamic drive for increased hepatic glucose production, lipid synthesis and mobilization, and insulin resistance. [9,10] When administered systemically [11-13] or into the cerebral ventricle [14] during the early hours of the light cycle, bromocriptine prevents or reverses seasonal fattening, insulin resistance and hyperinsulinemia, as well as decreases endogenous (hepatic) glucose production in mammals.

Cycloset™ is a new quick-release oral formulation of bromocriptine mesylate that has been employed in clinical studies to examine the effectiveness of timed bromocriptine administration to humans with metabolic disease including type 2 diabetes. Cycloset™ has been shown to reduce insulin resistance, glucose intolerance and hyperlipidemia during Phase II clinical trials. In three Phase III clinical trials, Cycloset™ significantly improved hyperglycemia among obese individuals with type 2 diabetes [15] Based on these study results, the FDA issued an approvable letter for Cycloset™ for the treatment of type 2 diabetes. Full approval is conditional, in part, on the completion of a large, placebo-controlled, randomized trial in persons with type 2 diabetes to evaluate fully drug-related adverse events with a high degree of certainty. The standard preparation of bromocriptine, Parlodel^®^, has been used for over 25 years in persons with Parkinson's disease, prolactinomas, acromegaly, infertility, and post-partum lactation, many of whom also had diabetes. However, there have been no prospective, randomized, placebo-controlled trials conducted which permit the estimation of incidence rate differences and ratios necessary for ruling out a clinically meaningful increased risk for adverse events in a population of persons with type 2 diabetes. This study will provide data that permits the estimation of serious adverse event incidence rate differences and ratios between Cycloset™ and placebo when these agents are added to usual diabetes therapy. In addition to the evaluation of safety parameters, we intend to evaluate pre-specified efficacy parameters among patient subgroups. Specifically we intend to assess changes in Hba1c among a subgroup of the study subjects that were not adequately controlled on metformin and sulfonylurea therapy. Clinical studies that combined Cycloset™ with metformin were not part of the original Cycloset™ clinical program because metformin was not commercially available in the United States at the time the studies were initiated.

## Methods/Design

### Analysis of safety

The primary objective of the study is to determine in subjects with type 2 diabetes receiving Usual Diabetes Therapy (UDT) and HbA1c ≤ 10%:

1. Whether add-on therapy with Cycloset™ results in all-cause rates of serious adverse events that are not higher than that of placebo by a pre-defined margin.

2. Whether add-on therapy with Cycloset™ results in disease-specific rates of serious cardiovascular adverse events that are not higher than that of placebo by non-inferiority margin of 1.5.

While the primary purpose of this study is to establish the safety profile of Cycloset™ in type 2 diabetes, any potential positive cardiovascular benefits will be evaluated as well.

### Analysis of efficacy

Hba1c changes from baseline to 24 weeks between Cycloset™ and Placebo among subjects with a baseline Hba1c of ≥ 7.5% among the following subgroups:

1. Treated at baseline with any Oral hypoglycemic agent (OHA) including injectable insulin secretagogues

a. Metformin plus or minus one OHA or injectable insulin secretagogue

b. Sulphonylurea plus or minus one OHA or injectable insulin secretagogue

2. Treated at baseline with Metformin and one sulphonylurea

### Study design and setting

This is a double blind, multi-center, placebo-controlled, parallel group, non-inferiority study in subjects with type 2 diabetes, comparing usual diabetes therapy (UDT) plus Cycloset™ to UDT plus placebo. Following a two-week lead-in period, 3,095 subjects were randomized in a 2:1 ratio to UDT plus Cycloset™ or UDT plus placebo across 73 clinical centers in the United States and Puerto Rico. Subjects were expected to remain on study treatment for 12 months, the first six weeks of which constitute a dose titration period.

#### Ethical considerations

Full ethical approval for this study was obtained. An external Data Monitoring and Safety Committee monitored the study progress.

#### Study interventions

The experimental condition is Cycloset^® ^in addition to the subject's current diabetes therapeutic regimen and lifestyle regimen. The control condition is placebo subject's current diabetes therapeutic regimen and lifestyle regimen. The regimen must consist of either diet, oral hypoglycemic agents (no more than 2), or insulin (alone or with no more than 1 oral hypoglycemic agent). The usual diabetes agents could not change during the first three months of the study. However, the dosages of the oral agents or insulin were modified as deemed appropriate by the study site investigator to achieve target glycemic goals recommended by the American Diabetes Association [16] After three months, alterations in the diabetes regimen were allowed if deemed necessary by the study site investigator to adhere to the ADA guidelines for achieving glycemic control. Prior to switching oral anti-diabetic medication to an alternative anti-diabetic medication, titration of the current oral anti-diabetic medication up to maximum tolerated dose was preferred and recommended to the study site investigators. However, the change in the regimen could not include additions that resulted in a final regimen that exceed two oral agents (not including study medication), or insulin, plus one oral agent (not including study medication).

#### Identification of eligible patients

Patients meeting the inclusion criteria were identified by research centers across the United States which included nineteen centers from the Veteran Affairs Healthcare System.

#### Confirmation of serious adverse events

Study site investigators elicited information regarding serious adverse events. The study safety officer reviewed all relevant safety data obtained from the study sites. Narratives were written from source documents. All serious adverse events (SAE) were verified for accuracy of coding for inclusion in the database. All SAEs were reviewed by an external adjudication committee consisting of three physicians (two cardiologists and one endocrinologist). Whenever possible, the medical records corresponding with the event were also reviewed.

#### Determining eligibility for the study

The principle investigator at each site reviewed the screening laboratories and medical history and assessed eligibility of screened study subjects (see table 1 inclusion and exclusion criteria).

**Table 1:** Inclusion/Exclusion Criteria

**Inclusion**	*Criteria*
Diagnosis Type 2 Diabetes	*≥ 6 months prior to enrollment*
Age	*30–80 years*
Hba1c at screening	*≤ 10*
Male or Female	*Female of child bearing age must use definitive contraceptive therapy*

***Exclusion***	

Taken Prescription sympathomimetic	*Taken seven days prior to screening*
Drugs not permitted during study	*Ergot alkaloid derivatives, or anti-migrane medications*
History of alcoholism or drug abuse	*Within 3 years of study entry*
Donation of blood	*30 days prior to study entry*
Has received any experimental drug or used an experimental device	*Within 30 days of study entry*
Pregnant or lactating women	
Known hypersensitivity to any of the formulation components	
***Subjects with clinically significant major organ system disease:***	
**• seizure disorder,**	
**• significant gastroparesis or orthostatic hypotension (autonomic neuropathy),**	
**• cerebrovascular accident in the previous 6 months,**	
**• uncontrolled hypertension (systolic BP >160 or diastolic BP > 100 at screening)**	
**• coronary artery bypass graft or coronary angioplasty in the previous 3 months, myocardial infarction in the previous 6 months, or unstable angina pectoris (chest pain at rest, worsening chest pain, or admission to the ER or hospital for chest pain) within the previous 3 months,**	
**• congestive heart failure defined by NYHA as Class III or IV**	
**• clinical nephrotic syndrome, or renal impairment with a serum creatinine > 1.4 mg/dl if female receiving treatment with metformin, > 1.5 mg/dl if male receiving treatment with metformin, and > 1.6 mg/dl in not on metformin,**	
**• impaired liver function, including having AST or ALT greater than three times the upper limit of normal,**	
**• active infection (e.g., HIV, hepatitis), or a history of severe infection during the 30 days prior to screening,**	
**• major surgical operation during the 30 days prior to screening,**	
**• cancer, other than non-melanoma skin or non metastatic prostate cancer within the past 5 years**	
**• Any concurrent illness, other than diabetes mellitus, not controlled by a stable therapeutic regimen.**	
**• Working rotating, varying or night shifts**	
**• Patients taking unapproved herbal supplements that may be associated with a risk of cardiovascular events (such as ephedra, yohimbe etc)**	
**• Patients who have started therapy with an erectile dysfunction drug within 2 weeks prior to screening; patients may not begin treatment with an erectile dysfunction drug during the study period; patients previously taking erectile dysfunction drugs should do so only under medical supervision.**	
**• Subjects with circumstances or abnormalities (e.g., blindness or a history of non-compliance) that would interfere with the interpretation of safety or efficacy data or completion of the study.**	

#### Patient follow-up procedures

After screening, patients were randomized and titrated to the maximum tolerated dose of the study drug over a six week period. After titration, subjects were seen every 3 months until study end (week 52) or early termination. Subjects were contacted 30 days after completion of study drug to record any adverse events occurring after treatment completion. See table 2 for visit schedule and assessments.

**Table 2:** Study Flow Sheet

			**STUDY WEEK**												
		-2	-1	0	1	2	3	4	5	6	12	24	36	52	Termination^3^

Assessment and Measurement	Pre-Screen	Screen	♠												

HbA1c: Local lab < 10	♣	♣													
Clinic Visit		X		X			X			X	X	X	X	X	X
Medical History		X													
Concomitant Meds and Illnesses		X					X			X	X	X	X	X	X
Pregnancy test*		X													
Full Physical Exam, *(Weight & Waist Circumference)*^1^		X										X		X	X
Blood pressure		XX		X			X			X	X	XX	X	X	X
Record Adverse events (AE)				X			X			X	X	X	X	X	X
ECG		X										X		X	X
Telephone Call to Subject for AE's Reports					X	X		X	X						
CBC & Plasma Sample		X										X		X	X
Chemistry, LAE♥		X^⊕^								X		X		X	X
HbA1c		X									X	X	X	X	X
Fasting Plasma Glucose^2^		X		X			X			X	X	X	X	X	X
Fasting Lipid Profile^♦2^		X										X		X	X

Notes on measures

♣ Local lab documentation of HbA1c ≤ 10 must be confirmed within 12 wks prior to conducting the Week-2 screening visit tests

♠ During Week-1 any labs or tests unable to be performed at screening should be completed

* To include pregnancy test (beta-HCG) in women of childbearing potential.

♥ CBC with differential and platelets, urinalysis, liver function (ALT, AST, alkaline phosphatase and total bilirubin), BUN, creatinine, Na, K, Cl, bicarbonate, calcium, phosphorus, and albumin.

♦ Total cholesterol, triglycerides, HDL-cholesterol, and LDL-cholesterol.

1. for body mass index and waist circumference to be measured on subject in the supine position at the level of the umbilicus

2. for BMI Obtained in the morning after a minimum 8 h overnight fast

3. A full 52-week assessment must be performed whenever a subject *terminates *the study early.

#### Patient outcome measures

##### Safety outcomes

Primary – all cause serious adverse events

Secondary – Composite of cardiovascular outcomes (myocardial infarction, hospitalized angina or congestive heart failure, stroke, or coronary revascularization)

##### Additional safety parameters

All adverse events, withdrawal due to an adverse event, laboratory values, vital signs and ECG

##### Other clinical measures

• Fasting lipid profile (total, LDL, HDL cholesterol, triglycerides) and

• HbA1c and

• Fasting plasma glucose level and

• Body weight, waist circumference, blood pressure

#### Sample size considerations

##### Sample size calculation for all-cause serious adverse events

In order to test the primary hypothesis at a one-sided alpha = .05 and have a power of 1-β = 0.90 when the non-inferiority margin is 1.5, the total number of subjects with all-cause serious adverse events that must be observed during the study is 235 with a sample size of 2,991 (assuming the placebo rate of 0.08).

##### Power calculation for serious cardiovascular adverse events

In order to test the hypothesis for serious cardiovascular adverse events as for the primary endpoint at a one-sided alpha = .05 when the non-inferiority margin is 1.5, the final sample size of 3000 to 3300 subjects will provide at least 62% power, assuming a hypothetical rate of events of 3.43%, or 103 to 113 cardiovascular serious adverse events.

##### Power calculation for efficacy analysis

With an expected 0.5% difference in mean change in HbA1c levels between the two treatment groups and a standard deviation of HbA1c level of 1.0% and the baseline and 24 week scores have a correlation of 0.50 then the effect size is 0.5%/1.0% = 0.50. For the metformin/sulphonylurea analysis, 160 subjects assuming a standard deviation of 1.0% will provide a power of 90% power to detect differences in mean changes of 0.5% or larger.

### Endpoints

#### Safety

• The primary safety endpoint is the rate of time to first treatment emergent all-cause serious adverse events.

• Secondary safety endpoints include disease-specific rate of time to first treatment emergent serious cardiovascular adverse events (myocardial infarction, stroke, inpatient hospitalization for heart failure or angina, and revascularization surgery) and the individual specific rates.

#### Efficacy

Other clinical measures include the impact (either positive or negative) of Cycloset™ on HbA1c, fasting plasma glucose, weight, triglycerides lipids, blood pressure and patient tolerability after six months of therapy. Inasmuch as concurrent diabetes medications may change over the 52 week period with greater probability than at 24 weeks, the primary efficacy analyses will be based on changes from baseline to 24 weeks of treatment.

### Statistical analysis – safety and other clinical measures

#### Safety

The Intention-To-Treat (ITT) analysis set will include all subjects who were randomized to the study and received at least one dose of study treatment (either Cycloset™ or placebo) and will be used for evaluating all safety endpoints. The Evaluable Per-Protocol (EPP) analysis set will consist of those ITT subjects with no major protocol violations and with at least one post-randomization measurement of HbA1c, fasting plasma glucose, lipids, weight, waist circumference or blood pressure. The primary analysis tested the hypothesis that the occurrence of all-cause serious adverse events for Usual Diabetes Therapy (UDT) plus Cycloset™ is not inferior to that for UDT plus placebo by a predefined margin. For this study, the predefined margin of non inferiority was defined as a hazard ratio (UDT plus Cycloset^® ^versus UDT plus placebo) of 1.5. The hazard ratio and one-sided 96% confidence interval will be obtained from the Cox regression model with treatment and center effects. The non-inferiority will be supported by the result that the upper bound of the confidence interval is below 1.5. The proportional hazards assumption underlying the Cox model will be examined with appropriate alternative statistical models applied. Results based on the ITT analysis set will be considered primary and those based on EPP analysis supportive. The secondary safety endpoint is the time to the occurrence of first cardiovascular SAEs (myocardial infarction, stroke, in-patient hospitalization for heart failure, angina or revascularization surgery). These endpoints will be analyzed in the same manner as described above for the primary endpoint

#### Other clinical measures

Clinical efficacy analyses will also be conducted for subjects taking any oral hypoglycemic agent (OHA) or injectable insulin secretagogue with a baseline HbA1c of ≥ 7.5%. Additional subsets of this population include those receiving a) metformin with or without any OHA or injectable insulin secretagogue and b) sulphonylurea with or without any OHA or injectable insulin secretagogue. The difference in the change in the HbA1c from baseline to 24 weeks between Cycloset™ and placebo will be the primary efficacy measure. The analyses will be based on both a) ITT – last observation carry forward method and b) subjects with an HbA1c measurement at week 24 (completers). An analysis of covariance (ANCOVA) model will be used to test for a treatment difference while adjusting for the baseline hemoglobin A_1c _value and center effect. Additional analyses will be used to assess the impacts from additional risk factors by including additional terms such as age, gender, body mass index at baseline, duration of disease history, and concomitant medications. Any significant effects from these potential risk factors will be further investigated. Analyses of treatment group differences in change from baseline in fasting plasma glucose, weight, waist circumference, triglycerides, lipid profile, and blood pressure will be explored within these defined groups.

We will also provide descriptive statistics on the changes in concomitant diabetes medications during the observational period (proportion of subjects adding therapy or increasing dose of preexisting therapy; proportion stopping therapy or decreasing dose; proportion not changing therapy). If warranted, additional analyses will assess the impact of such changes in concomitant diabetes medications during the observation period on difference between Cycloset™ and placebo in change from baseline in HbA1c.

#### Sub-study efficacy analyses

##### Metformin/sulfonylurea efficacy analysis

The Intention-To-Treat efficacy analysis set (ITTe) will include all subjects who were randomized to the study and at the time of screening were treated with metformin plus a sulfonylurea and had a screening HbA1c of ≥ 7.5%, and received at least one dose of study treatment (either Cycloset™ or placebo). The change in the HbA1c from baseline (screening visit week-2) to 24 weeks will be the primary efficacy measure. An analysis of covariance (ANCOVA) model will be used to test for a treatment difference while adjusting for the baseline hemoglobin A_1c _value and center effect. For those patients who discontinued prior to 24 weeks, the last post-randomization observation will be carried forward. The Evaluable Per-Protocol efficacy analysis (EPPe) set will consist of those ITT subjects with no major protocol violations and with an Hba1c measurement at 24 weeks. Analyses of treatment group differences in change from baseline in fasting plasma glucose, weight, waist circumference, triglycerides, lipid profile, and blood pressure will be explored.

Prior to un-blinding the study data it is not possible to consider all possible adjustments needed to fully develop the appropriate model for determining changes in various efficacy parameters. We will further explore and deploy the appropriate methods of statistical analysis to fully discern the differences between Cycloset™ and placebo in this large study.

## Competing interests

RS and AC are officers in the company Veroscience LLC which is developing Cycloset™ for the treatment of type 2 diabetes. ME is an employee of Veroscience. JG and DR report no competing interest involved with this trial.

## Authors' contributions

RS is responsible for writing, technical review and assurance of accuracy of all protocol sections. AC is responsible for writing and technical review. JG and ME are responsible for technical review. DR is responsible for technical review of statistical section. All authors reviewed and approved the final protocol.

## Pre-publication history

The pre-publication history for this paper can be accessed here:


